# Feasibility of Using the International Classification of Functioning, Disability and Health for Children and Youth (ICF-CY) as a Framework for Aquatic Activities: A Scoping Review

**DOI:** 10.3390/children10121856

**Published:** 2023-11-26

**Authors:** Merav Hadar-Frumer, Huib Ten-Napel, Maria José Yuste-Sánchez, Isabel Rodríguez-Costa

**Affiliations:** 1 Israel Sport Centre for the Disabled (ISCD) Ilan Spivak, Ramat Gan 52535, Israel; merav.hadar@edu.uah.es; 2 Faculty of Medicine and Health Sciences, University of Alcalá, 28807 Alcalá de Henares, Spain; marijo.yuste@uah.es; 3 WHO-FIC Collaborating Centre RIVM, 3720 Bilthoven, The Netherlands; huib.ten.napel@rivm.nl; 4 Department of Primary and Community Care, Radboud University Medical Centre, 6500 Nijmegen, The Netherlands

**Keywords:** the International Classification of Functioning, disability and health (ICF), disability and health: children and youth version (ICF-CY), aquatic activities, children aged 6–12, developmental delay, ICF-CY linking process

## Abstract

(1) Background: In recent years, reviewing studies of aquatic activities for children with developmental delays has been a complex task due to the multitude of indices and professional languages. (2) Aim: To determine if the ICF-CY framework can be used as the unifying language in AA studies of children with DD. (3) Methods: Part One—A systematic review of selected studies focusing on goals that were found to be positive. These goals were linked to the ICF-CY categories. Part Two—Review of all studies using the ICF-CY’s functioning components. (4) Results: Most of the positive goals were properly linked to ICF-CY and made it possible to review the 71 articles in a uniform language. (5) Conclusions: It is feasible to use the ICF framework as a universal structure and language.

## 1. Introduction

### 1.1. Aquatic Activities for Children with Developmental Delay

Within the aquatic environment (AE), aquatic activities (AAs) have been found to be effective for improving motor abilities, physical activity, social interaction, quality of life (QoL), and participation in children with developmental delay (DD), as well as activities of daily life and swimming skills [1,2,3,4,5,6,7,8,9,10,11]. In fact, the activity of children with DD in the aquatic environment is inseparable from their rehabilitation and treatment, and significantly enhances all areas of their life [12,13].

The recognition of the importance of activity in the AE is also reflected in the increase in the number of studies examining various AAs (such as swimming, aerobic activity, or therapy, individually or in a group) and their impact on children’s abilities and quality of life. Researchers investigated the effects of AA in different areas of life, such as activity and participation in daily life, changes in the body’s functions and structures, and the effect of the environment through the characteristics of the intervention or the physical conditions [1,2,3,4,5,6,7,8,9,10,11,12,13,14,15], and demonstrated the positive impact of AA on children with DD.

The fact that many different research professionals choose to investigate the effect of AA on children with DD is gratifying, and undoubtedly promotes the knowledge of professionals who work in this area. At the same time, the attempt to perform a systematic comparative analysis of the AA studies’ results is complicated by the numerous variations in local professional languages used by the researchers. This is exacerbated further by the many challenges that arise with the multitude of research goals and tools [15,16,17].

### 1.2. Possible Way Forward

To overcome the difficulties that arise when performing a systematic comparative analysis, many researchers refer to the International Classification of Functioning, Disability and Health (ICF), published by the World Health Organization (WHO) in 2001 [18], as the model that could serve as a unifying language and framework for pooling all the goals and tools [16,18,19,20]. This is because the ICF is a bio-psycho-social model which holistically includes a person’s functioning throughout his/her life and unites the medical approaches with the social approaches in one framework.

### 1.3. Background Rational for Using the ICF as a Unifying Framework and Language

**Figure 1:**
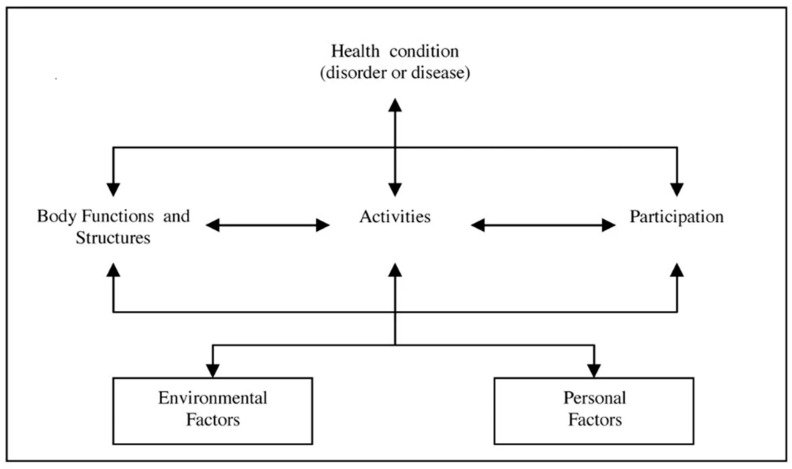
The ICF framework: interaction between ICF components. Adapted from [18,21,22].

The ICF provides a framework and standard language, as well as a description of health and health-related states. According to the ICF framework, a person’s state of health is not the only factor that determines his/her everyday abilities and functioning. Rather, it is one of six different components—Health condition, Body Functions (BF) and Body Structures (BS), Activities (A), Participation (P), Environmental Factors (EF), and Personal Factors (PF)—all of which dynamically interact with each other. A change in any of these factors will have an impact on the others [18,21,22] (Figure 1).

The ICF was originally intended to provide a classification covering the lifespan of functioning for all human beings without differences in religion, race, sex, or age. However, after several years of experience working with the ICF framework and standard language, the experts working with children concluded that the ICF is not detailed enough to assess children’s abilities, especially in areas related to age and development, and does not consider their dependence on others and their living conditions. As a result, the ICF-children and youth (ICF-CY) was developed and launched in 2007, which provides more detailed items regarding children and youth, mainly in the areas of A&P [22,23].

The structure of the framework, its holistic nature, and its ability to provide a uniform language are the reasons that many researchers recommend it as the model that provides professionals in the fields of AA the means to investigate, formulate goals, and communicate in a uniform and comprehensible manner [9,15,18,20,21,24,25,26,27]. To promote the link between the ICF and the world of research, and to enable a link between the various professional languages and the ICF language, Cieza and her partners [16,17] developed guidelines for the linking procedures. These guidelines have been tested in various studies and are recommended for use in the processes in which the researchers connect and unify the various research elements in the terms of the ICF model [21,25]. These guidelines allow researchers to choose the ICF framework as a unifying language and a holistic tool for reviewing studies, examining research results, or evaluating measurement tools. The following discusses some examples.

Güeita-Rodríguez and his partners [8] were looking for the types of intervention that aquatic physical therapists (APTs) choose to promote with children having different limitations. To this end, they asked APTs from around the world what areas they believe are related to the functioning of these children, including contextual factors. The answers were subjected to an agreement process using the Delphi technique and a linking procedure to the ICF-CY. At the end, a preliminary list of the various categories of intervention within the AE treatment for these children was defined. This research, and additional preliminary research they conducted [27], eventually led to the presentation of a list of core sets for AA with children and youth having neurological limitations, which aims to help researchers and professionals in the field to set quality goals for treatment and research [28].

Schiariti et al. [29] examined the results of various studies of children with CP to determine whether the results of the studies could be described by the ICF-CY categories (via a link to the ICF-CY), and which areas the researchers could use the most, in relation to functioning. The researchers found that, through the link to the ICF-CY, it was possible to provide a detailed content analysis, and thus allow the professionals and researchers to adapt their outcome measures to the intended purpose. The ICF-CY link identifies the measures tested and provides new information about how to characterize each measure based on the ICF categories.

Adolfsson et al. [30] examined the involvement of children in need of special support in preschool using the Child Engagement Questionnaire (CEQ). They examined, utilizing the link to the ICF-CY framework, whether the questionnaire is holistic enough and provides comprehensive information on the children’s degree of involvement and participation in kindergarten. The researchers chose the ICF-CY comparison because it is a model that provides information on all the child’s functions in a comprehensive and holistic way. They believe that linking the items of the CEQ to the ICF-CY codes will provide an understanding about the instrument’s structure—is it sufficiently holistic and does it cover enough aspects to assess the child engagement and participation in preschool? Their conclusions were as follows: (1) ICF-CY can be applied in early childhood research in areas of child involvement, despite challenges in the ICF-CY definitions that need to be addressed, for example, challenges regarding children’s involvement in play and ways to separate playing from activities related to learning through play. (2) CEQ does not provide the information required for a sufficiently holistic understanding of the child’s involvement in kindergarten; it covers some areas related to ACT and PAR but does not refer at all to areas related to body functions or the environment.

Björklund et al. [31] reviewed the records of professionals who cared for children who had been rehabilitated after recovering from brain tumors. Their goal was to compare these records to the ICF categories to unify all the reports (of healthcare professionals and educators) into one language that describes the children’s daily difficulties in the areas of body functions, activity, and participation. The researchers found that it is possible to make this link and use the ICF categories for documentation purposes. At the same time, difficulties arose in connecting the various challenges manifested in the body, functioning, participation in daily life, and education, all of which are crucial to obtain a holistic picture of the child’s condition, into a “connecting network of problems”.

### 1.4. What Is Known about the Extent to Which Linking Rules Are Used in Studies of AA for Children?

In the field of AA, as in other fields of treatment, there is increasing awareness of the importance of linking to the ICF-CY framework and language, with the aim of defining a universal taxonomy of intervention goals for the professionals working in AE with children [8,27,28]. Along with this increase in awareness, there is a limited number of studies that facilitate the necessary linking procedure between the local professionals’ language and the ICF terms [15].

In a scoping review performed earlier by the authors of the current article [32], we found that nine of all the articles that had investigated the effect of AA on children with DD between the years 2010 and 2020 carried out a linking procedure between the research results and the ICF’s model terms. In a significant portion of these nine articles, the exact method of the linkage process was not very clearly described. In others, there was only a partial explanation of the methodology. Only one group of authors—Güeita-Rodríguez et al. [8,27,28]—provided a full description of the recommended methodology while developing five preliminary aquatic physical therapy (APT) ICF core sets for children. Their articles significantly promoted the linkage between the ICF framework and the AA. Questions that arose from these studies were related to the fact that the researchers relied on the opinion of experts (with a consensus procedure) regarding the effects of APT and did not use evidence about the effectiveness of the aquatic interventions from previous studies [33].

Considering the lack of comparability of studies raised above, in the current literature review we identified the important ICF components and the prominent categories that were studied in the various articles on the effect of AA on children with DD, while reviewing the articles that were found to be relevant.

### 1.5. The Main Aim of This Study

The main aim of this study is to determine if the ICF-CY framework can be used as the unifying language in AA studies of children with DD, and to determine whether the ICF-CY can be used as the main tool for researchers and professionals in the field of AA for assessment and setting goals. This will provide a homogeneous language of the studies among the professionals and a basis for comparisons and links between the various research reports.

To achieve this objective, we would like to examine it in two ways:

(A) Assessing the feasibility of linking—To examine whether the goals found to be positive in the selected articles can be linked to the language of the ICF-CY.
(B) Reviewing the articles—To examine whether it is possible to review the results of the relevant articles with the unifying language of the ICF-CY framework.

A positive conclusion in these two steps will lead to the conclusion that the ICF-CY framework language can act as a unifying language of the various assessment tools and serve as an alternative to these tools.

## 2. Materials and Methods

For the present systematic review, we followed the principles of the PRISMA-Scr (Preferred Reporting Items for Systematic reviews and Meta-Analyses extension for Scoping Reviews) Checklist [34]

### 2.1. Study Design

The research procedure consisted of three stages:

(1) Collecting all the appropriate studies between 1.1.2010 and 31.1.2020 and the selection of articles that met the inclusion/exclusion criteria established by the researchers.
(2) Reviewing the selected articles for all the positive results.
(3) Carrying out a linking process of all the “positive goals” to the ICF-CY framework.
(4) A systematic review of the selected articles using the language of the ICF-CY framework.

### 2.2. Search Strategy

#### 2.2.1. Article Selection

General selection definitions: For the systematic review, studies published in the period from 1 January 2010 to 31 January 2020 and which investigated the effect of AA on children with DD in elementary school age (6–12 years-old) were collected.

The search was limited to studies published in English, full articles, and articles open to the public on the internet or in the medical libraries of Alcala University, Be’er Sheva University, Tel Aviv University, and Sheba Medical Centre. The keyword combinations used for the search were the term “A child/children” with all terms related to aquatic activity and accompanying the following concepts: “hydro”, “aquatic”, “pool”, “swimming” and “water” (Table 1).

#### 2.2.2. Electronic Databases

Relevant articles were identified by searching the international healthcare databases: PubMed, PubMed Central^®^ (PMC), Google Scholar, Physiotherapy Evidence Database (PEDro), Cochrane Library, Researchgate, Scientific Research, and Scielo. The search also reviewed the bibliographic references of the collected papers for the purpose of locating additional studies not found in the basic database.

#### 2.2.3. Inclusion and Exclusion Criteria

To select the appropriate studies, a screening process was performed by two of the researchers (MHF and IRC). Each reviewer went through all the articles independently. Then, lists were compiled and compared, and a procedure was implemented in which articles were agreed upon. The screening procedure relied on the criteria selected by all researchers as being appropriate for this study. The main areas that were defined were the following four:

Study characteristics: We were looking for articles that would illustrate new insights into the topic of the research. Therefore, they had to be full articles that contained all the details of the research conducted, including the full results, and articles that allowed the researchers to discover new findings in the field. This includes types such as descriptive research, systematic scoping reviews, literature reviews, intervention reviews, narrative reviews, quasi-experiments, and integrative reviews. Articles published on a private website, by commercial organizations, or on company websites were not included.

Main population: The main population on which the study was conducted were children with developmental delays/disorders, aged 6–12 years old. This age group was selected because children of these ages tend to have similar developmental characteristics and a defined social stage—the elementary school—with similar learning abilities and social requirements—referred to as “middle childhood” [35], and thus can be adapted to similar functioning and participatory goals.

Aquatic methods used in the interventions: Since this review focuses on studies that examine the effect of AA and the AE on children, we chose articles in which the researchers focused on AA as the main variable of the study, or those in which the effect of the unique aquatic environment was selected as a factor influencing the children’s activity, in combination with a familiar device from land.

We included flotation supportive tools because these devices are unique to the AE and are effective due to the up-thrust force (a unique feature for this environment). No other additional aquatic accessories were included. The intervention types included different aquatic activities such as swimming, therapy, or any other physical activity in the water, performed individually or in groups. The techniques used by the instructors (a unifying term for all professionals who guide or treat children in the AE according to the Halliwick approach [1,14]), the means of instruction, and the nature of its accessibility to the children, as well as the surrounding environment in which the intervention took place, were different and diverse.

### 2.3. Analyzing and Linking Processes

#### 2.3.1. Article Screening and AA Goal Selection

To link the study’s results to the ICF-CY’s domains, a careful process of reviewing the selected studies was performed by two of the researchers (IRC, MHF). Within the process, all the goals found to be positive (“positive results”) in each research result were selected, as well as all the measurement tools used by the researchers in the various studies.

#### 2.3.2. The ICF-CY Linking Process

To link all the positive treatment goals to the ICF-CY (English version), the researchers applied the “refined ICF linking rules” published by Cieza et al. 2019 [16]. The first part of the data analysis was carried out by the two researchers (MHF, HTN). The linking process itself involved eight discussion stages (a “critical appraising consensus process”), and was carried out as follows:

Step One—All the positive results from the reviewed articles were collected and written in a verbal version of the article itself.

Step Two—The lead researcher (MHF) examined all the results and linked them according to the linking rules, and to the various domains, codes, and categories of the ICF-CY.

Step Three—The ICF and ICF-CY expert (HTN) examined all the links made by MHF and performed one of the following options: (a) approved the link; (b) asked for clarification regarding the choice of a particular category; or (c) objected to the specific choice and offered another option.

Step Four—MHF examined all the comments and corrected or responded with explanations and a rational for a selection to HTN.

Steps Five–Eight—Discussions continued between the two researchers until there was full agreement on all links.

### 2.4. A Short Systematic Review

A systematic review was carried out of the selected articles using components, domains, and categories from the ICF-CY framework.

## 3. Results

### 3.1. Articles Identification

**Figure 2:**
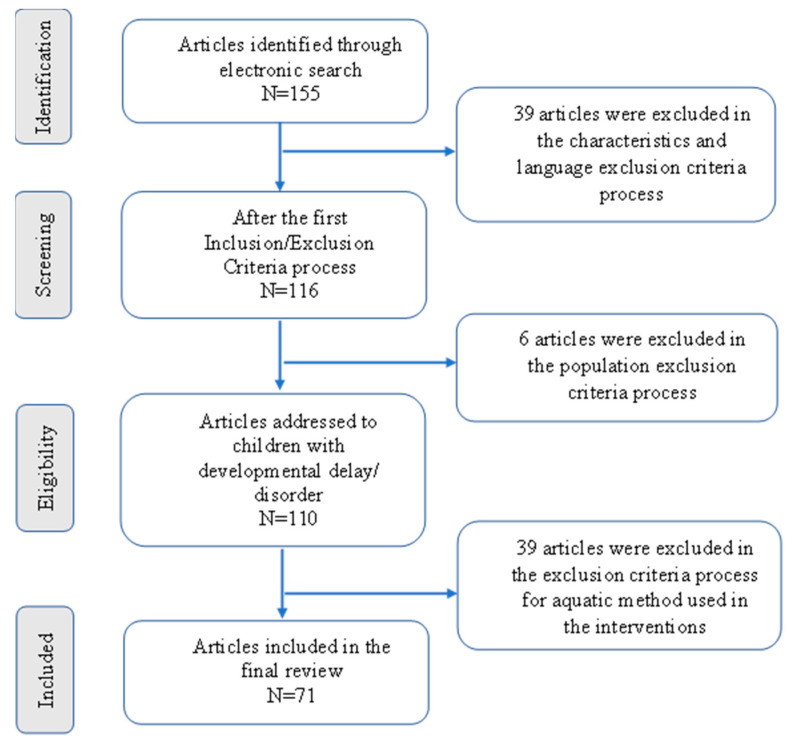
Search review flowchart (initial search was completed in January 2020).

After the first screening, 155 papers which met with the initial criteria were listed. The next screening process involved a careful review of all the articles, and the subsequent selection of eligible articles based on the inclusion/exclusion criteria that are included in the study, i.e., characteristics, population, comparators, and aquatic methods. After reviewing all the papers, 84 were excluded and 71 were then included for the review (Figure 2 and Table A1 in Appendix A).

### 3.2. Results of the Analysis

#### 3.2.1. Article Screening and Selection of AA Positive Results

In the process of extracting the data from the various studies, we extracted the following topics: (1) main health condition/diagnosis; (2) intervention methods; (3) assessment tools used by the researchers; and (4) intervention goals that were found to have a positive effect in the study.

##### The Various Health Conditions/Diagnoses

The 71 selected studies examined the effect of the AA on 24 different types of health conditions/diagnoses which were grouped into ten groups, according to the main health conditions/diagnoses investigated in the studies:

(1) Cerebral palsy (CP) [27,36,37,38,39,40,41,42,43,44,45,46,47,48,49,50,51,52,53,54,55,56,57];
(2) Autistic spectrum syndrome (ASD) [7,10,58,59,60,61,62,63,64,65,66,67,68,69,70,71,72,73,74,75,76,77];
(3) Different developmental delays (DDs)—the DD group included all the studies that examined several disorders together in the same study—CP, Paresis, Spina Bifida/Myelomeningocele (SB/MMC), ASD, Down Syndrome, DD, Nonverbal Learning Disorder, Oto-Palatal-Digital Syndrome, Central and Peripheral Neurological Disorders, Psychomotor Delay, Musculoskeletal Disorders, ADHD, Noonan Syndrome [6,8,14,78,79,80,81];
(4) Muscular dystrophy diseases (Mus. D)—DMD—Duchenne muscular dystrophy and CMD—congenital muscular dystrophy [82,83,84,85,86,87];
(5) General health conditions (GHCs)—a group that included Asthma, Hemophilia, Juvenile Dermatomyositis, and Obesity [88,89,90,91,92];
(6) Juvenile Idiopathic Arthritis (JIA) [93,94,95];
(7) Attention Deficit Hyperactivity Disorder (ADHD) [96,97];
(8) Development Coordination Disorder (DCD) [9];
(9) Down Syndrome [98];
(10) Rett Syndrome [99].

##### Intervention Methods

In general, most of the interventions focused on activities with individuals. There were some studies that examined the effect of AA in group activities. Most of the programs used one type of intervention, with only a few programs mixing several interventions.

The most extensively used intervention (28 studies) was defined by the authors of the reviewed articles as “conventional AT” (aquatic therapy techniques without a specific definition), most of which were conducted in a one-on-one activity [6,7,33,34,35,36,37,38,39,42,51,55,57,63,69,70,72,74,78,80,82,84,85,86,87,88,91,92,93,97,99,100]. In two articles the researchers defined their intervention as “aquatic physical therapy” (APT) [8,27]. The use of well-known AT approaches was mainly based on the Halliwick approach (20 studies) in various ways, i.e., swimming, treatment, playing individually or in a group [9,10,14,40,41,43,44,45,46,49,50,52,60,62,63,71,73,75,76,77,79,83], with one study testing the effects of the Watsu technique along with conventional AT [96]. Twelve studies based their research on swimming or on the promotion of swimming abilities, including various swimming learning programs and the adapted swimming exercises, in groups or individuals [7,50,59,64,65,79,81,89,90,93,98,99]. Four studies examined the effect of walking, running, and aerobic activity in the AE on the participants’ functioning [47,48,93,94], and nine other studies examined eight different special therapeutic programs in the AE, with the aim to promote sensation, strength, fitness, communication, and social relations [53,54,56,58,61,66,67,68,95].

##### Assessment Tools

Overall, 132 different measuring instruments were found to be used by the researchers to identify the effect of the AA on the functioning of the children who participated in the studies (Table A2 in the Appendix A).

##### Positive Intervention Results

A total of 443 different positively formulated intervention goals were collected for the ICF-CY linking process.

### 3.3. The ICF-CY Linking Process Results

#### 3.3.1. The ICF-CY Categories

In the Linking Process, the 443 Positive AA Treatment Goals Found in the Articles Were Linked to 270 ICF-CY Categories.

The categories were divided into the four following groups of components: (1) Activity and Participation (A&P)—138 categories that were extracted in the linking process from 470 different ICF-CY links; (2) Body Functions (BF)—98 categories that were extracted from 397 different ICF-CY links; (3) Environment factors (EF)—22 categories from 42 different ICF-CY links; and (4) Body Structures (BS)—12 categories from 20 different ICF-CY links. In the various studies, the personal factors (PF) of the participants were not mentioned; therefore, this important component does not appear in the tables.

In the diagnostic groups of CP and DD, references were found to the four different components—BF, BS, A&P, and EF. Within the ASD group, there was no reference to the BS component, and within the other diagnostic groups in the studies, references were found only to the BF and A&P components.

Table 2 shows the distribution of the categories produced in the ICF-CY linking process according to the 10 different diagnostic groups. The categories are divided in accordance with the ICF’s components. The list of all categories linked in the review is presented in Table A3, Table A4, Table A5 and Table A6 within Appendix A.

#### 3.3.2. The Most Used Components and Categories of the ICF-CY

In the data obtained, it was found that the researchers in the various studies referred to all of the components of the ICF model except for the PF component. In components BF, BS, and A&P, the chapters that were most widely used in the various studies were the chapters that referred to movement and mobility, whilst the chapter referring to support and relationships was the most widely used in the environment components. Swimming was the most frequently used category in the studies—it was found to be positive 21 times. The components used by the researchers in the various studies, as well as the prominent category in each domain, can be seen in Table 3.

## 4. Discussion

Regarding our main objective, i.e., to determine if the ICF-CY framework can be used as the unifying language in AA studies of children with DD, several issues emerge. The ICF linking process with the ICF as a unifying language for the systematic review proved to be useful for unification of different studies with different measurement tools and different professional languages, thus enabling the production of consolidated results for a literature review. However, the process also proved to be very complex and showed the limitations within the linking process when only looking at the ICF and its framework because of the multiplicity of tools in the different studies. This is explained next.

The main limitations that arose in the process of linking to the ICF-CY are as follows:

The number of measuring tools—The researchers in the selected articles used 132 different measuring tools to learn about the effects of the AA on the children’s functioning (Table A2 in the Appendix A). Each measuring tool has its own special definitions and professional language it represents. In order to perform the linking process of the different intervention goals to the ICF-CY, we focused on the intervention goal itself and not on the measurement tool.

By choosing such an action, we were able to harmonize the language among all the studies we reviewed and to use the ICF-CY tool as a unifying language framework.

The study found limitations and shortcomings in the structure and content of the ICF-CY framework itself. We experienced significant difficulties while trying to link the ICF-CY language to a number of important concepts such as QoL, activities of daily living (ADL), different behaviors, or changes in health status, as described below:

- Quality of life is a very broad concept and is a very important subject in every person’s life [23]. According to the WHO, the definition of QoL depends on the perception of each person of their position in life within the context of their environment, such as their culture, the value systems they were raised under, and their standards, all of which are in relation to the person’s own life goals [22,25,100,101]. This important concept still does not have a structured and clear definition in the model. Thus, in our attempts to link different positive goals from the studies that referred to QOL, we had to expand each individual goal and identify the specific area of quality of life that the authors referred to in their article. The authors used many different tools, for example, the Cerebral Palsy Quality of Life Questionnaire for Children (CPQOL) [43], Health-related quality of life—HRQOL [81] or the Short Form-36 items (SF-36), and the Burn Specific Health Scale Brief (BSHS-B) [102].

Regarding the Personal Factor and well-being, these two important concepts also have no precise definitions or elaboration within the ICF-CY framework [25,103,104]. In recent years, few articles have been published offering classifications and definitions for the PF component.

In 2019, Threats, et al. [105] presented an option for the ICF’s PF definitions. In their work they formulated a classification based on the principles of the ICF framework which represents the “lived experience of health from the personal factors perspective” [106] (p. 1732) of persons with spinal cord injury. The classification contains seven areas and four hierarchical levels. In 2019, Geyh, et al. [106] also published their revisited personal factor classification, i.e., The German Society for Social Medicine and Prevention (DGSMP) classification of personal factors, which had five chapters with definitions, categories, explanations, and inclusions/exclusions.

The researchers in both articles emphasize the importance of defining the PF within the ICF framework as for all other components. They stress that this kind of upgrade is a way to focus on the individual in every area related to his/her health condition, i.e., treatment, research, and policy making. Doing so provides everyone with better and more personalized service and support.

In their review from 2021, Grotkamp, et al. [107] examined all the categories related to PF and rehabilitation that appeared in 226 selected articles. The researchers recommend the classifications developed by Threats, et al. [105] or Geyh, et al. [106] as the primary checklists for the next investigations [107].

- The definition of well-being in the framework of the ICF is very short and concise—“Well-being is a general term encompassing the total universe of human life domains, including physical, mental and social aspects, that make up what can be called a “good life” [22] (p. 227)”. Although this is a very important concept, there is not much reference to it in the model; references are only in areas related to health and health systems, and not in areas of employment, education, etc.

When examining a person’s subjective sense of well-being, researchers link the individual’s personal characteristics, such as values, spirituality and religion, satisfaction from work, and behavior, with his subjective well-being (SWB), and emphasize the importance of the referrals of professionals and researchers to the personal factor as an integral part of a person’s SWB [108,109].

- The goals connected to activities of daily living were very difficult to link. ADLs are defined as “tasks that are fundamental to supporting participation across school, home and community environments” [110] (p. 223). In the categories of the ICF-CY framework, each of these activities is defined separately within the components of “Activities and Participation”. There is no specific reference to this definition of functioning as a whole. For example, Zanobini and Solari [77], referred to the “self-help skill” goal from the ABC questionnaire. The areas they referred to focused on independence in toilets, eating, drinking, and dressing. To relate this goal to ICF-CY, it was necessary to refer to eight different ICF-CY categories.
- Changes over time or due to interventions for a different health status or in various bodily functions, such as pain, muscle tone, etc., are impossible to link. Terms such as “improved”, “increased”, “more”, and “severity”, which indicate changes in the condition over time or intervention, do not have clear scales and definitions within the ICF-CY model [25,101]. For a linking process to be possible in these cases, in their study, the researchers must use the qualifiers which can offer information about the amount of change. Without specified qualifiers, these terms have no clear meaning in the ICF-CY framework. In addition, changes in movement characteristics such as in gait analysis, i.e., speed, stride length, dynamic balance, etc., do not have appropriate definitions within the ICF-CY framework.

Finally, but also difficult, are goals that were related to behavioral characteristics, such as autistic symptoms, and belong to the mental domains, which are not defined within the ICF-CY framework, but in the ICD and DSM-V. 

All these intervention goals were impossible to link and, therefore, are also not listed in the table (Table A3, Table A4, Table A5 and Table A6 in Appendix A).

Regarding the second objective, the following brief review presents the possibility for researchers in the fields of AA for children with DD to use concepts from the ICF-CY framework in order to gather the various articles and reach common conclusions. 

In this study we reviewed 71 different articles that examined the effect of AA on children with DD. These studies focused on 10 main groups of health conditions only. Within the 10 groups there were a total of 24 different health conditions, the vast majority of which focused on children with CP (23 studies [27,36,37,38,39,40,41,42,43,44,45,46,47,48,49,50,51,52,53,54,55,56,57]) and ASD (22 studies [7,10,58,59,60,61,62,63,64,65,66,67,68,69,70,71,72,73,74,75,76,77]).

It is the writer’s opinion, based on experiences in working with children in AA, that many more types of populations and health conditions/diagnoses do benefit from AA, with the purpose of promoting the child’s QoL, functioning, and social and personal abilities. 

Overall, the various studies had a broad reference to most of the ICF components except for the PF. Within the various diagnostic groups studied, a prominent trend could be observed that there is a relationship between the examined health condition and the studied ICF-CY components. The studies that examined the effect of AA on children with ASD [7,10,58,59,60,61,62,63,64,65,66,67,68,69,70,71,72,73,74,75,76,77] focused more than the others on the A&P component; out of 125 categories that were linked to the positive results, 76 were related to this component. That is, 60.8% of the positive results referred to areas from the A&P component, compared to studies on children with CP (48.5%) [27,36,37,38,39,40,41,42,43,44,45,46,47,48,49,50,51,52,53,54,55,56,57], or compared to the studies on children with DD (46.7%) [6,8,14,78,79,80,81]. The impression from these data is that researchers who study the effect of the AA on children with ASD view the activity in the AE as an opportunity to promote all parts of the child’s everyday-life abilities. As such, they focus their research far more on the participation goals of daily life, compared to other researchers who examine the effect of AA on children with physical disabilities. This last group refer to the changes in body functions as goals, which are more or less of equal importance as the goals regarding the children’s participation.

At the same time, it is interesting to note the fact that only the studies on children from the CP and DD diagnostic groups referred to four components of the ICF-CY (except for PF), and their research goals were very numerous and varied. The field of research on children with DD is particularly notable since, despite the relatively small number of studies (only 7 studies compared to 23 studies on children with CP and 22 studies on children with ASD), the researchers found 167 different categories with a positive effect of the intervention; this was the second largest number among the 10 diagnostic groups (after the CP children, with 175 categories).

The remainder of the researchers who studied all the other diagnostic groups chose fewer goals and only addressed the components of A&P and BF. We believe that the reason for these notable differences stems from two factors: (a) The DD group is a very diverse group with many different health conditions (compared to the other groups in which there was much more uniformity in diagnoses) and, as such, the researchers had to expand the range of areas examined in each study. (b) The other research groups (besides CP and ASD) do not have much previous research and prior knowledge; therefore the researchers focus mainly on areas where the effects of AA are better known and recognized.

Another important thing we would like to address is the two components that are part of the ICF framework within the contextual factors:

(a) The environment component: A gratifying finding was that environment as a whole became part of the areas which the researchers refer to, and especially areas related to the AE, the family, the friends, and the social connections. We found categories from the EF component only in the CP, ASD, and DIS health groups. Within the studies that examined the effect of AA on these three groups, 18 articles [6,8,14,28,33,37,39,43,45,47,49,52,53,57,65,74,75] found 22 different environmental categories that had a positive effect on children with DD who participated in AA. These 22 positive ICF-CY categories were extracted from all five chapters of this component (see Table A5 in Appendix A). The prominent areas were supports and relationships, and mainly focused on the categories related to the health professionals. Since the AE has unique properties that differ from those of land [111], it would make sense that the researchers would refer to this important issue in their studies. The AA techniques used in the studies were very diverse and included therapy, various functional activities, and participation. AA can be undertaken individually or in a group, in a controlled environment (therapy pool), or in a community pool, with therapists, friends, or family members. It is gratifying that the main reference in all studies was not only to the physical effects of this environment, but to the vital social aspects. This fact indicates that the researchers attach importance to AE as a factor affecting the social ability of children with DD.
(b) The personal factor—A disturbing finding was the lack of reference to the children’s personality characteristics. These characteristics were mentioned in few studies, but were not examined at all as intervention goals in the study. The researchers Güeita-Rodríguez and associates [8,27,28] explained that their decision to ignore this component in their studies was due to the fact that it had not yet been classified in the ICF-CY. Fragala-Pinkham and her colleagues [47] recommended examining this area in future studies. Ballington and Naidoo [39] mentioned this component together with EF as factors that may influence children’s ability to participate in physical activity, but did not refer to it later in their article.

Considering the fact that several options have been proposed for defining personal characteristics in accordance with the ICF framework [105,106], and that studies have already been conducted for testing the use of these specifications to analyze the results of studies and to link them to the PF factor [107], it is the opinion of this article’s authors that the PF component is very important in all cases of an intervention affecting the child’s life. How PF should be handled is an ethical question. We stipulate that these personal characteristics should be agreed upon by the person themselves or by their proxy.

We concur with the opinion of researchers Ferguson et al. [112] and Ueda and Okawa [113], who call on the professionals to always refer to the contextual factors as part of all the components they examine in the research, since these are factors that affect the children’s abilities (whether they are facilitators or barriers), and any change in them over time will affect all other domains of the children’s functioning.

The following components and domains were very prominent in the studies:

(a) Movement and mobility—Within the wide variety of positive intervention goals, one can see from the results (Table 3) that the most frequently used categories in all the ICF-CY components were categories related to movement and mobility. The functions of the neuromuscular-skeletal and movement systems (BF), the structures related to movement (BS), and the mobility (A&P) chapters were the most frequently used chapters in the studies. These findings are not surprising, since the activity in the AE is considered to be an activity that stimulates movement and provides good balance control, due to AE properties such as buoyancy, up-thrust, and hydrostatic pressure [111]. This enable activities to be experienced that are sometimes very difficult on land for children with DD [4,5]. The properties of the AE, such as density, viscosity, and turbulence, along with the temperature of the water in the therapeutic pool (usually 32–34 °C), allows work on strengthening, cardiopulmonary endurance, and improving range of motion without much physical load on the skeleton and joints, and without the risk of falling [111].
(b) Swimming—Notably, the field of swimming was the category that was tested the most in the various studies (21 times; Table 3). Swimming is a very important activity and a participation factor for children with developmental delays [44,114], both as a social and health factor. As Stubbs [115] concluded in his review from 2017: “Swimming remains one of the most popular forms of physical activity across the world and may offer a unique opportunity to promote, maintain and improve wellbeing across the lifespan, with potential to reach all individuals of society, regardless of gender, age, disability or socioeconomic status.” [115] (p. 27).

### 4.1. A Summary of the ICF-CY Review

From this review it can be concluded that AA has an effect on the functions of children with DD in broad areas of their daily life. Evidence was found for the effect of AA in many areas of the children’s A&P components and, at the same time, positive effects were found on other areas such as BF and the environment in which the child acts. The researchers point to swimming as an important tool for promoting all areas of the children’s daily lives.

### 4.2. Recommendations of the ICF-CY Review

The various health conditions/diagnoses groups—We recommend that future studies expand the range of health conditions/diagnoses investigated and evaluate the effect of AA on children with additional health conditions, such as emotional impairment or developmental intellectual disability. We also recommend the broadening of the level of knowledge of health conditions such as respiratory diseases, orthopedic impairments, metabolic diseases, attention disorders, and muscle diseases.

Further development of the ICF-CY—In terms of the linking difficulties that arose in the current study, within the ICF-CY framework itself, we recommend that the developers of the framework address the issues that were found to be difficult to link, and give them definitions within the ICF-CY language to enable researchers to use them as ICF-CY goals in future studies. It is important to mention that both the ICF and the ICF-CY state in the chapter that indicates the tasks for the future the importance of developing these areas alongside other areas—“developing a component of personal factors, and creating connections with perceptions of quality of life and the measurement of subjective well-being” [18] (p. 251), [22] (p. 264)].

### 4.3. Limitations of Our Research and Recommendations for Future Research

The main difficulty in the process of linking the positive goals from the various studies to the ICF-CY categories stemmed from the fact that there are still not many studies in the field and, therefore, without examples from previous linking procedures, the linking procedure in this study was a very complex task. We would recommend that researchers in the future who study the effect of AA on children with DD create agreed links of the well-known measurement tools, such as the Gross Motor Function Classification System (GMFCS), the Life Inventory Quality of Life (PedsQL), and Water Oriented Test Alyn (WOTA), to the ICF-CY language, thereby facilitating the process of linking and analyzing the results of studies in this field.

## 5. Conclusions

This study offers a synthesis review of the intervention goals found to be positive in aquatic activity. From the linking process of the positive research goals, it can be concluded that it is feasible to use the ICF Framework as a universal structure and language that allows the following:

(a) Combining the different research studies’ results into a review with joint results and conclusions.
(b) Promoting the uniformity of the outcome measures of the studies (when using the ICF-CY), which will enable researchers to examine the interrelationship between all interventions’ elements, and identify important domains within the AA goals of interventions.
(c) Implementing the changes that apply among the children in terms of the various functioning components (i.e., BF, BS, A&P, EF, and PF), within the unique aquatic environment.
(d) Using the ICF-CY language as a unifying factor between the various professionals working with the child in AA.

From a practical point of view, the research indicates the ambiguity of the terminology used, which hinders the collection of a body of evidence in an organized manner. We have shown that there is much overlap and similarity in the terminology used. Using ICF-CY terminology offers the advantage of possible pooling of data, even afterwards. Our message is to use ICF-CY or ICF for curriculum design and content.

Our review also demonstrates the limitations within the process of linking to the ICF and offers proposals that will allow solving the problem of the multiplicity of tools in the different studies, using the framework and language of the ICF-CY.

The authors of this article support the opinions expressed in previous articles, and recommend the development and implementation of domains within the ICF model for important subjects such as QoL and well-being, ADL, and PF.

We recommend that future studies put more emphasis on the aquatic environment itself as a meaningful and important environment within the social and personal contexts of all children with DD, regardless of their health condition (or type of disability).

## Figures and Tables

**Table 1:** Initial search criterion—key words.

Hydrotherapy Aquatic Therapy Aquatic Activities Aquatic Exercise Aquatic Exercise Programs Aquatic Fitness Aquatic Group Therapy Aquatic Physical Therapy Aquatic Programs Aquatic Sports Aquatic-Based Exercise Program Aquatics Aerobic Aquatic Gymnastics Pool Therapy Pool Therapy Method Swimming Swimming Rehabilitation Swimming Therapy Swimming Training Water Activities Water Based Exercise Water Environment Water Exercise Water Immersion Water Therapy	and	A child Children

**Table 2:** The ICF-CY categories linked from the positive goals according to the different diagnostic groups.

The Investigated Diagnosis	CP	ASD	DD	Mus. D	GHC	JIA	ADHD	DCD	Down S.	Rett S.
Number of Articles—out of the total 71 chosen articles	23	22	7	6	5	3	2	1	1	1
2. Activity & Participation categories (Number of different categories used in each diagnostic group)	85	76	78	13	1	1	3	3	0	12
3. Body Functions categories (Number of different categories used in each diagnostic group)	68	45	66	9	8	5	8	4	1	6
4. Environment categories (Number of different categories used in each diagnostic group)	14	4	11	0	0	0	0	0	0	0
5. Body Structures categories (Number of different categories used in each diagnostic group)	8	0	12	0	0	0	0	0	0	0
6. Total use of ICF categories in the diagnostic group	175	125	167	22	9	6	11	7	1	18

ADHD—Attention Deficit Hyperactivity Disorder, ASD—Autistic spectrum syndrome, CP—Cerebral palsy, DCD—Development Coordination Disorder, DD—Developmental Delay, Down S.—Down Syndrome, GHC—General health conditions, JIA—Juvenile Idiopathic Arthritis, Mus. D—Muscular dystrophy diseases, Rett S.—Rett syndrome.

**Table 3:** The most common components and categories in the various studies.

ICF-CY Component	BF	BS	A&P	EF
1. Chapters that were used	All eight chapters	Chapters s2, s3, s4, s7 and s8	All but Chapter 6	All five chapters
2. The most frequently used chapter (the N. of times its categories have been used)	b7—Neuromusculoskeletal and movement-related functions (183)	s7—Structures related to movement (14)	d4—Mobility (258)	e3—Support and relationships (16)
3. The most used category (the N. of times it has been used)	b755—Involuntary movement reaction functions (17)	There was no prominent category	d4554—Swimming (21)	e355—Health professionals (10)
4. Health conditions/diagnoses that tested the most of the categories in this component (N. of times)	CP (206)	DD (12)	CP (237)	CP (23)

A&P—Activities and Participation, BF—Body Functions, BS—Body Structures, EF—Environment Factor, ICF-CY—The International Classification of Functioning, Disability and Health for Children and Youth.

## Data Availability

Not applicable.

## Appendix A

**Table A1:** The characteristics of the articles studied (arranged in alphabetical order).

**First Author and Year (Alphabetical Order)**	**Main Subject**	**Ages**	**Sample and Size**	**Aquatic Activity**	**Research** **Design**
Adar, S. et al., 2017 [36]	CP	4–17	32	Conventional AT	RCT
Akinola, B.I. et al., 2019 [37]	CP	1–12	30	Conventional AT	RCT
Alaniz, M.L. et al., 2017 [58]	ASD	3–7	7	Group therapy	QE, NCG
Aleksandrović, M. et al., 2010 [78]	Neuromuscular Impairments—CP, Paresis, SB	5–13	7	Adapted swimming program, Halliwick	QE, NCG
Badawy, W.M. et al., 2016 [38]	CP	6–9	30	Conventional AT	RCT
Ballington, S.J. & Naidoo, R. 2018 [39]	CP	8–12	10	Halliwick	RCT
Bayraktar, D. et al., 2019 [93]	JIA	11–16	42	Water-running program	QE, NRCT
Birkan, B. et al., 2010 [59]	ASD	8–9	3	Halliwick	QE, NCG
Caputo, G. et al., 2018 [60]	ASD	6–9	23	Multisystem Aquatic Therapy	QE, NRCT
Carew, C. & Des, W.C. 2018 [88]	Asthma	9–16	41	Swimming	RCT
Chang, Y.K. et al., 2014 [96]	ADHD	5–10	30	Conventional AT—group	QE, NRCT
Christodoulaki, E. et al., 2018 [40]	CP	5–15	8	Halliwick	QE, NCG
Chu, C. & Pan, C. 2012 [61]	ASD	7–12	42 (21 ASD + 21 TD)	Halliwick	QE, NRCT
De la Cruz, R.D. & Robert, D. 2012 [41]	CP	7–17	4	Conventional AT	QE, NCG
Declerck, M. et al., 2013 [43]	CP	5–13	7	Halliwick	QE—A pilot study, NCG
Declerck, M. 2014 [44]	CP	7–17	14	Swimming intervention—Halliwick	RCT- A pilot study
Declerck, M. et al., 2016 [42]	CP	7–17	14	Swimming intervention—Halliwick	RCT
Dimitrijevic, L. et al., 2012 [45]	CP	5–14	27	Swimming intervention—Halliwick	RCT
Elnaggar, R. & Elshafey, M.A. 2016 [94]	JIA	8–11	30	Combined resistive underwater exercises and interferential current therapy	RCT
Ennis, E. 2011 [62]	ASD	3–9	6	Conventional AT, Halliwick	QE, NCG
Fatorehchy S. et al., 2019 [46]	CP	6–9	6	Walking aquatic therapy program	QE, NCG
Ferreira, A.V.S. et al., 2015 [82]	DMD	8–24	23	Halliwick	QE retrospective study, NCG
Fragala-Pinkham, M. et al., 2010 [6]	Different disabilities—ASD, CP, DS, MMC, DD, NLD, OPTS	6–12	16	Pilot aquatic exercise program—swimming and Conventional AT	QE, NCG
Fragala-Pinkham, M.A. et al., 2011 [7]	ASD	6–12	12	Group aquatic exercise program—conventional AT	QE, NRCT
Fragala-Pinkham, M.A. et al., 2014 [47]	CP	6–15	8	Aquatic aerobic exercise program—individual	QE, NCG
Güeita-Rodríguez, J. et al., 2017 [8]	Children with Disabilities—ND, ASD, PMD, MD	children	--	Aquatic physical therapy	EP (interview)
Güeita-Rodríguez, J. et al., 2018 [27]	CP	0–18	34 parents	Aquatic physical therapy	EP (interview)
Hamed, S.A. & Fathy, K.A. 2015 [89]	Hemophilia	7–10	30	Swimming exercise program	QE, NCG
Hamed, S.A. et al., 2016 [98]	DS	8–12	30	Swimming training program, conventional AT	RCT
Hillier, S. et al., 2010 [9]	DCD	5–8	12	Halliwick	RCT
Hind, D. et al., 2017 [83]	DMD	7–16	at the end—9	Standardized AT	QE, single-blind RCT, with nested qualitative research
Honório, S. et al., 2013 [84]	DMD	9–11	7	Conventional AT	QE, NCG
Honorio, S. et al., 2016 [85]	DMD	9–11	3	Conventional AT	QE, NRCT
Ilinca, I. et al., 2015 [79]	Children with Disabilities—DS, ASD, CP	10–16	6	Conventional AT	QE, NCG
Jorgić, B. et al., 2014 [48]	CP	6–17	15	Halliwick, backstroke swimming	QE, NCG
Jorgić, B. et al., 2012 [49]	CP	8–10	7	Halliwick, swimming	QE, NCG
Jull, S. & Mirenda, P. 2016 [63]	ASD	5–8	8	Swimming	QE, NCG
Kafkas, A.S. & Gökmen, Ö.Z.E.N. 2015 [64]	ASD	8	1	Swimming	QE, NCG, A Pilot Study
Kim, K.H. & Hwa, K.S. 2017 [50]	CP	3–7	20	Conventional AT	QE, one group pretest-posttest design
Kim, K.H. et al., 2018 [80]	Different Disabilities—not detailed	5–20	10	Swimming exercise program	QE, NCG
Lai, C.J. et al., 2015 [51]	CP	4–12	24	Halliwick	QE, NRCT
Lawson, L.M. & Little L, 2017 [67]	ASD	5–12	10	Sensory Enhanced Aquatics	QE, NCG
Lawson, L.M. et al., 2019 [65]	ASD	4–18	14 children; 14 Parents	Family water activities	QE, NCG
Lawson, L.M. et al., 2014 [66]	ASD	4–18	42	Sensory-supported swimming^®^ lessons	QE, NCG
Maniu, D.A. et al., 2013 [52]	CP	8–16	24	Aquatic therapy program	QE, NCG
Maniu, D.A. et al., 2013 [53]	CP	8–16	24	Conventional AT	QE, NCG
Wadu Mesthri, S. 2019 [56]	CP	7–11	5	Conventional AT	QE, pretest-posttest design, NCG
Mills, W. et al., 2020 [68]	ASD	6–12	8	Conventional AT	RCT, A Pilot Trial
Olama, K.A. et al., 2015 [54]	CP	5–7	30	Conventional AT	RCT
Oriel, K.N. et al., 2016 [69]	ASD	6–11	8	Conventional AT	QE, NCG, A Pilot Study
Oriel, K.N. et al., 2012 [81]	Different Disabilities—SB, ADHD, CP, ASD, NS	7–18	23	Conventional AT	QE, NCG
Pan, C.Y. 2010 [10]	ASD	6–9	16	Halliwick	QE, controlled single-blind design
Pan, C.Y. 2011 [70]	ASD	7–12	15 with ASD, 15 siblings	Halliwick	QE, NRCT
Pushkarenko, K. et al., 2016 [71]	ASD	11–17	3	Conventional AT	QE, An interrupted time series design (A/B/A), A Pilot Study
Ramírez, N.P. et al., 2019 [95]	JIA	8–18	46	Watsu and conventional AT	RCT
Ryu, K. et al., 2016 [55]	CP	8–48 (aquatic group—9–13)	32	Assisted aquatic movement	RCT
Salem, E.Y. et al., 2016 [90]	Asthma	6–12	40	Conventional AT	QE
Samhan, A. et al., 2020 [91]	Juvenile Dermatomyositis	10–16	14	Conventional AT	QE, A 2 × 2 Controlled-Crossover Trial
Santos, C.P.A. et al., 2016 [86]	CMD	6	1	Conventional AT	QE, NCG, A case study
Shams-Elden, M. 2017 [72]	ASD	8–11	10	Halliwick therapy	QE, NCG, A case report
Silva, K.M. 2012 [87]	DMD	12	1	Conventional AT	QE, NCG, A case study
Silva, L.A.D. et al., 2020 [97]	ADHD	11–14	20	Swimming–learning program	RCT
Stan, A.E. 2012 [92]	Overweight	5–8	7	Conventional AT, running, swimming	QE, NCG
Torres, L.E. et al., 2019 [99]	Rett syndrome	4–7	3	WaterFit MITAF program (Integral Method of Functional Aquatic Work)	QE, NCG, A case report
Vaščáková, T. et al., 2015 [14]	Severe Disabilities—CP, ASD	4–7	10	Halliwick	QE, NCG
Wilson, K.E. 2019 [73]	ASD	4–13	6	Conventional AT	QE, NCG
Yanardag, M. et al., 2013 [74]	ASD	6–8	3	Aquatic play skills intervention—based on Halliwick	QE, NCG
Yanardag, M. et al., 2015 [75]	ASD	6	3	Halliwick	QE, NCG
Yilmaz, I. et al., 2010 [76]	ASD	9	3	Halliwick	QE, NCG
Zanobini, M. & Solari, S. 2019 [77]	ASD	3–8	25	“Water as a Mediator of Communication” program	QE
Zverev, Y. & Kurnikova, M. 2016 [57]	CP	5–17	13	Community-based group aquatic program—aimed to balance	QE, NCG

ADHD—Attention Deficit Hyperactivity Disorder; ASD—Autism Spectrum Disorder; AT—Aquatic Therapy; CMD—Congenital Muscular Dystrophy; CP—Cerebral Palsy; DCD—Development Coordination Disorder; DD—Developmental Delay; DMD—Duchenne Muscular Dystrophy; DS—Down Syndrome; EP—Experts Opinion; JIA—Juvenile idiopathic arthritis; MD—Musculoskeletal Disorders; MMC—Myelomeningocele; NCG—No control group; ND—Central and Peripheral Neurological Disorders; NLD—Nonverbal Learning Disorder; NRCT—Non-Randomized Control Trial; NS—Noonan Syndrome; PMD—Psychomotor Delay; QE—Quasi-experiment; RCT—A Randomized Controlled Trial; SB—Spina Bifida; TD—Typical Development.

**Table A2:** List of measurement tools used in the studies.

	**Measurement Tools**	**CP**	**ASD**	**DMD+ CMD**	**DD**	**Health Con.**	**JIA**	**ADHD**	**DCD**	**Down Syn.**	**Rett Syn.**	**Total**
1	A Gima Oxy-4 oximeter	1	0	0	0	0	0	0	0	0	0	1
2	A goniometer	2	0	0	0	0	0	0	0	0	1	3
3	A sleep log	0	1	0	0	0	0	0	0	0	0	1
4	A Spirometer	1	0	0	0	2	0	0	0	0	0	3
5	A survey for experts	0	0	0	1	0	0	0	0	0	0	1
6	Ability to increase and maintain swimming skill	0	1	0	0	0	0	0	0	0	0	1
7	activity limitations measure (ACTIVLIM)	0	0	1	0	0	0	0	0	0	0	1
8	American Red Cross learn-to-swim levels	0	1	0	0	0	0	0	0	0	0	1
9	Anthropometric circumference measurements										1	1
10	Aquatic skills checklist (ASC)	0	1	0	0	0	0	0	0	0	0	1
11	Balance Master System	0	0	0	0	0	0	0	0	1	0	1
12	Barthel ADL Index	0	0	0	0	0	0	0	0	0	1	1
13	Basic Motor Ability Test-Revised (BMAT)	0	0	0	0	0	0	1	0	0	0	1
14	Biodex balance system	1	0	0	0	0	0	0	0	0	0	1
15	Biodex Gait Trainer	1	0	0	0	0	0	0	0	0	0	1
16	blood collection	0	0	0	1	0	0	0	0	0	0	1
17	Brockport Physical Fitness Test (BPFT)	1	0	0	0	0	0	0	0	0	0	1
18	Carefussion PulmoLife spirometer	2	0	0	0	0	0	0	0	0	0	2
19	Carer quality of life (CarerQoL)	0	0	1	0	0	0	0	0	0	0	1
20	Cerebral Palsy Quality-of-Life–parent proxy scale (CP QoL—parent)	1	0	0	0	0	0	0	0	0	0	1
21	Child Depression Inventory (CDI)	0	0	0	0	0	0	1	0	0	0	1
22	Child Health Utility 9D Index (CHU9D)	0	0	1	0	0	0	0	0	0	0	1
23	Childhood Autism Rating Scale (CARS)	0	2	0	0	0	0	0	0	0	0	2
24	Children’s Assessment of Participation and Enjoyment (CAPE	1	0	0	0	0	0	0	0	0	0	1
25	Children’s OMNI Scale of Perceived Exertion (OMNI RPE)	0	0	1	0	0	0	0	0	0	0	1
26	Children’s Sleep Habits Questionnaire (CSHQ)	0	1	0	0	0	0	0	0	0	0	1
27	Compliance test	0	1	0	0	0	0	0	0	0	0	1
28	Computerized Evaluation Protocol of Interactions in Physical Education (CEPI-PE)	0	1	0	0	0	0	0	0	0	0	1
29	Demographic form	0	1	0	0	0	0	0	0	0	0	1
30	document investigation and observation	0	1	0	0	0	0	0	0	0	0	1
31	EKS—Egen Klassifikation Scale	0	0	4	0	0	0	0	0	0	0	4
32	Electrically-braked cycle ergometers	0	0	0	0	0	1	0	0	0	0	1
33	Electroencephalograms	1	0	0	0	0	0	0	0	0	0	1
34	electromyography (EMG)	0	0	1	0	0	0	0	0	0	0	1
35	Energy Expenditure Index (EEI)	0	0	1	0	0	0	0	0	0	0	1
36	Enjoyment regarding the swimming intervention	1	0	0	0	0	0	0	0	0	0	1
37	forced vital capacity (FVC)	0	0	1	0	0	0	0	0	0	0	1
38	Functional Independence Measure (FIM)	0	0	0	0	0	0	0	0	0	1	1
39	functional reach test (FRT)	0	0	1	0	0	0	0	0	0	0	1
40	Health and social care resource-use questionnaire	0	0	1	0	0	0	0	0	0	0	1
41	health-related quality of life (HRQoL)	1	0	0	0	0	0	0	0	0	0	1
42	Heart rate										1	1
43	Hoffman reflex	1	0	0	0	0	0	0	0	0	0	1
44	HUMAC NORM—Isokinetic Dynamometer	0	0	0	0	0	1	0	0	0	0	1
45	Humphries’ Assessment Of Aquatic Readiness (HAAR)	0	5	0	0	0	0	0	0	0	0	5
46	Imagery Rehearsal Therapy (IRT)	0	1	0	0	0	0	0	0	0	0	1
47	Isometric muscle strength	0	0	0	0	1	0	0	0	0	0	1
48	Jamar Hydraulic Hand Dynamometer	1	0	0	0	0	0	0	0	0	0	1
49	Jebsen-Taylor Hand Function test	1	0	0	0	0	0	0	0	0	0	1
50	Kinemtaic gait parameters	1	0	0	0	0	0	0	0	0	0	1
51	Life Habits Short Form questionnaire (LIFE-H)	1	0	0	0	0	0	0	0	0	0	1
52	Lung pressures	0	0	1	0	0	0	0	0	0	0	1
53	Measures of program acceptability and safety	1	0	0	0	0	0	0	0	0	0	1
54	Mercury sphygmomanometer	0	0	0	1	0	0	0	0	0	0	1
55	Metabolic Gas Analysis Systems	0	0	0	0	0	1	0	0	0	0	1
56	Modified Ashworth Spasticity (MAS)	3	0	0	0	0	0	0	0	0	0	3
57	Movement Assessment Battery for Children—Second Edition (Movement M-ABC, ABC-2)	0	1	0	0	0	0	0	1	0	0	2
58	multidimensional fatigue scale (MFI)	1	0	0	0	0	0	0	0	0	0	1
59	North Star Ambulatory Assessment (NSAA)	0	0	1	0	0	0	0	0	0	0	1
60	0ne—minute fast walk test	3	0	0	0	0	0	0	0	0	0	3
61	Paediatric Escola Paulista de Medicina Range of Motion Scale (pEPM-ROM)	0	0	0	0	0	1	0	0	0	0	1
62	Patient Global Assessment (PGA)	0	0	0	0	1	0	0	0	0	0	1
63	Pediatric Balance Scale (PBS)	3	0	0	1	0	0	0	0	0	0	4
64	Pediatric Evaluation of Disability—PEDI (PEDI-NL; M-pedi; PEDI-CAT)	2	1	0	0	0	0	0	0	0	0	3
65	Pediatric Reach Test (PRT)	1	0	0	0	0	0	0	0	0	0	1
66	Peer Sociometric Nomination Assessment (Friendship Questionnaire)	0	0	0	1	0	0	0	0	0	0	1
67	Percentage of fat mas	0	0	1	0	0	0	0	0	0	0	1
68	Physical Activity Enjoyment Scale scores (PACES)	1	0	0	0	0	0	0	0	0	0	1
69	Physical Activity Index	1	0	0	0	0	0	0	0	0	0	1
70	Pictorial Scale of Perceived Competence and Social Acceptance (PSPCSA)	0	0	0	0	0	0	0	1	0	0	1
71	Piers-Harris 2 Children’s Self-Concept Scale	0	0	0	1	0	0	0	0	0	0	1
72	Pool tests—20 m run, the standing broad jump test, Mushroom Float, and Walking	0	1	0	0	0	0	0	0	0	0	1
73	Program satisfaction—evaluation questionnaire for parents/children	1	3	0	1	0	0	0	0	0	0	5
74	Quality of Life Questionnaire for Children (CP QoL-Child)	1	0	0	0	0	0	0	0	0	0	1
75	Questionnaire for measuring quality of life in children and adolescents (KINDLR)	1	0	0	0	0	0	0	0	0	0	1
76	Questionnaire on Parent’s Perception of Changes in their Child’s Participation	0	0	0	0	0	0	0	1	0	0	1
77	School Social Behavior Scales (SSBS–2)	0	1	0	0	0	1	0	0	0	0	2
78	Semi-structured interviews and focus groups	1	1	0	0	0	0	0	0	0	0	2
79	Sensory Profile Caregiver Questionnaire	0	2	0	0	0	0	0	0	0	0	2
80	Shuttle Run Test (SRT-I & SRT-III)	1	0	0	0	0	0	0	0	0	0	1
81	six-min walk distance (6 MWD)	0	0	1	0	0	0	0	0	0	0	1
82	Six-Minute Walk Test (6 MWT)	1	0	0	0	0	0	0	0	0	0	1
83	Skin Disease Activity Score (Dasskin)	0	0	0	0	1	0	0	0	0	0	1
84	Skinfolds										1	1
85	Social and ecological validity survey	0	1	0	0	0	0	0	0	0	0	1
86	Social Responsiveness Scale, 2nd edition (SRS-2)	0	2	0	0	0	0	0	0	0	0	2
87	Social Skills Improvement System (SSIS)	0	1	0	0	0	0	0	0	0	0	1
88	Social validity—parent questionnaires	0	1	0	0	0	0	0	0	0	0	1
89	Spatial-temportal gait variables	1	0	0	0	0	0	0	0	0	0	1
90	Sustainability of the aquatic exercise program	0	0	0	1	0	0	0	0	0	0	1
91	Swimming Classification Scale (SCS)	1	1	0	1	0	0	0	0	0	0	3
92	Swimming skill acquisition	0	1	0	0	0	0	0	0	0	0	1
93	Swimming With Independent Measure (SWIM)	1	0	0	0	0	0	0	0	0	0	1
94	Ten-joints Global range of motion score (GROMS)	0	0	0	0	0	1	0	0	0	0	1
95	Ten-meter walking speed (10-MWT)	2	0	0	0	0	0	0	0	0	0	2
96	The 16-m modified PACER	0	0	0	1	0	0	0	0	0	0	1
97	The amount of training time required to achieve a skill	0	1	0	0	0	0	0	0	0	0	1
98	The anaerobic-to-aerobic power ratio	0	0	0	0	0	1	0	0	0	0	1
99	The Autism Behavior Checklist (ABC)	0	1	0	0	0	0	0	0	0	0	1
100	The Borg Rating of Perceived Exertion (RPE)	0	0	0	0	1	0	0	0	0	0	1
101	The Bruininks-Oseretsky Test of Motor Proficiency (BOT)	1	0	0	0	0	0	0	0	0	0	1
102	The Carefussion MicroPeak flow meter	1	0	0	0	0	0	0	0	0	0	1
103	The Child Behaviour Checklist (CBCL)	0	1	0	0	0	0	0	0	0	0	1
104	The Feeling Scale	1	0	0	0	0	0	0	0	0	0	1
105	The Felt Arousal Scale (FAS)	1	0	0	0	0	0	0	0	0	0	1
106	The Gross Motor Function Measure (GMFM-88/GMFM-66)	6	0	0	1	0	0	0	0	0	0	7
107	The half mile walk/run	0	1	0	0	0	0	0	0	0	0	1
108	The International Physical Activity Questionnaire—IPAQ—parents	0	1	0	1	0	0	0	0	0	1	3
109	The Korean-trunk control measurement scale (K-TCMS)	1	0	0	0	0	0	0	0	0	0	1
110	The Körperkoordinations Test für Kinder (KTK)	0	0	0	0	0	0	1	0	0	0	1
111	The modified curl-up and isometric push-up tests	0	1	0	0	0	0	0	0	0	0	1
112	The Motor Function Measurement (MFM)	0	0	1	0	0	0	0	0	0	0	1
113	The National Physical Fitness Survey	0	0	0	1	0	0	0	0	0	0	1
114	The Pediatric Quality of Life Inventory quality of life (PedsQL-CP)	3	1	0	1	1	1	0	0	0	0	7
115	The Perceived Stress Scale (PSS)	0	0	0	0	0	0	1	0	0	0	1
116	The Progressive Aerobic cardiovascular fitness (PACER)	0	1	0	0	0	0	0	0	0	0	1
117	The sit-and-reach test	0	0	0	0	0	0	1	0	0	0	1
118	the TAC Cancellation Attention Test	0	0	0	0	0	0	1	0	0	0	1
119	The Timed Up and Go test (TUG)	3	0	0	0	0	0	0	0	0	0	3
120	The Trail Making Test (TMT)	0	0	0	0	0	0	1	0	0	0	1
121	The Ventilatory Function Tests (VFTs)	0	0	0	0	1	0	0	0	0	0	1
122	The Vignos scale	0	0	1	0	0	0	0	0	0	0	1
123	The Visual Analogue Scale (VAS) and the Faces Pain Scale (FPS-R)	2	0	1	0	0	2	0	0	0	0	5
124	The Wee Functional Independence measure (WeeFIM)	1	0	0	0	0	0	0	0	0	0	1
125	Timed Up and Down Stairs (TUDS)	1	0	0	0	0	0	0	0	0	0	1
126	Verbal evaluation Test	0	0	0	1	0	0	0	0	0	0	1
127	Vineland Adaptive Behavior Scales (VABS)	1	1	0	0	0	0	0	0	0	0	2
128	Water Oriented Test Alyn (WOTA)	6	1	0	2	0	0	0	0	0	0	9
129	Weight and BMI	0	0	1	0	1	0	0	0	0	0	2
130	Wingate Test	0	0	0	0	0	1	0	0	0	0	1
131	YMCA Water Skills Checklist	0	1	0	0	0	0	0	0	0	0	1
132	Zigzag agility test	0	0	1	0	0	0	0	0	0	0	1
	Total Measurements	74	45	22	17	9	11	7	3	1	7	196

ADHD—Attention Deficit Hyperactivity Disorder, ASD—Autistic spectrum syndrome, CP—Cerebral palsy, DCD—Development Coordination Disorder, DD—Developmental Delay, Down S.—Down Syndrome, JIA—Juvenile Idiopathic Arthritis, Rett S.—Rett syndrome.

**Table A3:** The list of all ICF-CY categories found in the review (*—categories which the authors of this article feel that, in order to make them meaningful, they need a qualifier). Body Functions (98 categories).

**N.**	**ICF-CY Code**	**ICF-CY Category**	**N.**	**ICF-CY Code**	**ICF-CY Category**	**N.**	**ICF-CY Code**	**ICF-CY Category**
1	b110	Consciousness functions	34	b176	Mental function of sequencing complex movements	67	b5253	Faecal continence
2	b114	Orientation functions	35	b180	Experience of self and time functions	68	b530	Weight maintenance functions
3	b1143 *	Orientation to objects	36	b210	Seeing functions	69	b6202	Urinary continence
4	b117	Intellectual functions	37	b2300 *	Sound detection	70	b710	Mobility of joint functions
5	b122	Global psychosocial functions	38	b235	Vestibular functions	71	b7101	Mobility of several joints
6	b125	Dispositions and intra-personal functions	39	b2350	Vestibular function of position	72	b715	Stability of joint functions
7	b1250	Adaptability	40	b2351	Vestibular function of balance	73	b720	Mobility of bone functions
8	b1252	Activity level	41	b2352	Vestibular function of determination of movement	74	b730	Muscle power functions
9	b1254	Persistence	42	b250	Taste function	75	b7300	Power of isolated muscles and muscle groups
10	b126	Temperament and personality functions	43	b255	Smell function	76	b7301	Power of muscles of one limb
11	b1263	Psychic stability	44	b260	Proprioceptive function	77	b7302	Power of muscles of one side of the body
12	b1264	Openness to experience	45	b265	Touch function	78	b7303	Power of muscles in lower half of the body
13	b1266	Confidence	46	b270	Sensory functions related to temperature and other stimuli	79	b7304	Power of muscles of all limbs
14	b130	Energy and drive functions	47	b2703	Sensitivity to a noxious stimulus	80	b7305	Power of muscles of the trunk
15	b1301	Motivation	48	b280	Sensation of pain	81	b7306	Power of all muscles of the body
16	b1304	Impulse control	49	b2800	Generalized pain	82	b735	Muscle tone functions
17	b134	Sleep functions	50	b310	Voice functions	83	b740	Muscle endurance functions
18	b1340	Amount of sleep	51	b330	Fluency and rhythm of speech functions	84	b750	Motor reflex functions
19	b1342	Maintenance of sleep	52	b410	Heart functions	85	b755	Involuntary movement reaction functions
20	b1344	Functions involving the sleep cycle	53	b415	Blood vessel functions	86	b760	Control of voluntary movement functions
21	b140	Attention functions	54	b420	Blood pressure functions	87	b7600	Control of simple voluntary movements
22	b144	Memory functions	55	b4302	Metabolite-carrying functions of the blood	88	b7601	Control of complex voluntary movements
23	b147	Psychomotor functions	56	b435	Immunological system functions	89	b7602	Coordination of voluntary movements
24	b1470	Psychomotor control	57	b440	Respiration functions	90	b7603	Supportive functions of arm or leg
25	b1471	Quality of psychomotor functions	58	b4401	Respiratory rhythm	91	b7608	Control of voluntary movement functions, other specified- Half km/4 points
26	b152	Emotional functions (happiness)	59	b4402	Depth of respiration	92	b761	Spontaneous movements
27	b1520	Appropriateness of emotion	60	b445	Respiratory muscle functions	93	b7611	Specific spontaneous movements
28	b1522	Range of emotion	61	b450	Additional respiratory functions	94	b765	Involuntary movement functions
29	b156	Perceptual functions	62	b455	Exercise tolerance functions	95	b7653	Stereotypies and motor perseveration
30	b160	Thought functions	63	b4550	General physical endurance	96	b770	Gait pattern functions
31	b163	Basic cognitive functions	64	b4552	Fatiguability	97	b780	Sensations related to muscles and movement functions
32	b164	Higher-level cognitive functions	65	b510	Ingestion functions	98	b840	Sensation related to the skin
33	b1643	Cognitive flexibility	66	b525	Defecation functions			

ICF-CY—The International Classification of Functioning, Disability and Health: Children and Youth Version.

**Table A4:** Activity and Participation (138 categories).

**N.**	**ICF-CY Code**	**ICF-CY Category**	**N.**	**ICF-CY Code**	**ICF-CY Category**
1	d110	Watching	71	d430	Lifting and carrying objects
2	d115	Listening	72	d4302	Carrying in the arms
3	d120	Other purposeful sensing	73	d435	Moving objects with lower extremities
4	d1201	Touching	74	d4351	Kicking
5	d1202	Smelling	75	d440	Fine hand use
6	d1203	Tasting	76	d4400	Picking up
7	d129	Purposeful sensory experiences, other specified and unspecified	77	d4402	Manipulating
8	d130	Copying	78	d445	Hand and arm use
9	d131	Learning through actions with objects	79	d4452	Reaching
10	d1310	Learning through simple actions with a single object	80	d4454	Throwing
11	d132	Acquiring information	81	d4455	Catching
12	d135	Rehearsing	82	d450	Walking
13	d137	Acquiring concepts	83	d4500	Walking short distances
14	d140	Learning to read	84	d4501	Walking long distances
15	d145	Learning to write	85	d4508	Walking, other specified—walking in water
16	d155	Acquiring skills	86	d455	Moving around
17	d159	Basic learning, other specified and unspecified	87	d4550	Crawling
18	d160	Focusing attention	88	d4551	Climbing
19	d1601	Focusing attention to changes in the environment	89	d4552	Running
20	d161	Directing attention	90	d4553	Jumping
21	d170	Writing	91	d4554	Swimming
22	d175	Solving problems	92	d4555	Scooting and rolling
23	d177	Making decisions	93	d4558	Moving around, other specified -walking backwards/KN walk/with hand support
24	d210	Undertaking a single task	94	d460	Moving around in different locations
25	d2103	Undertaking a single task in a group	95	d465	Moving around using equipment
26	d220	Undertaking multiple tasks	96	d469	Walking and moving, other specified and unspecified
27	d2203	Undertaking multiple tasks in a group	97	d510	Washing oneself
28	d230	Carrying out daily routine	98	d520	Caring for body parts
29	d2300	Following routines	99	d530	Toileting
30	d2302	Completing the daily routine	100	d540	Dressing
31	d2303	Managing one’s own activity level	101	d550	Eating
32	d2304	Managing changes in daily routine	102	d560	Drinking
33	d240	Handling stress and other psychological demands	103	d570	Looking after one’s health
34	d2401	Handling stress	104	d571	Looking after one’s safety
35	d250	Managing one’s own behavior	105	d598	Self-care, other specified
36	d2500	Accepting novelty	106	d599	Self-care, unspecified
37	d2504	Adapting activity level	107	d710	Basic interpersonal interactions
38	d310	Communicating with—receiving—spoken messages	108	d7101	Appreciation in relationships—satisfaction
39	d3101	Comprehending simple spoken messages	109	d7102	Tolerance in relationships
40	d3102	Comprehending complex spoken messages	110	d71041	Maintaining social interactions
41	d315	Communicating with—receiving—nonverbal messages	111	d7105	Physical contact in relationships
42	d325	Communicating with—receiving—written messages	112	d7106	Differentiation of familiar persons
43	d330	Speaking	113	d720	Complex interpersonal interactions
44	d331	Pre-talking	114	d7200	Forming relationships
45	d332	Singing	115	d7202	Regulating behaviors within interactions
46	d335	Producing nonverbal messages	116	d7203	Interacting according to social rules
47	d3352	Producing drawings and photographs	117	d730	Relating with strangers
48	d345	Writing messages	118	d740	Formal relationships
49	d350	Conversation	119	d7400	Relating with persons in authority
50	d3500	Starting a conversation	120	d7402	Relating with equals
51	d355	Discussion	121	d750	Informal social relationships
52	d410	Changing basic body position	122	d7504	informal relationships with peers
53	d4100	Lying down	123	d760	Family relationships
54	d4101	Squatting	124	d7600	Parent-child relationships
55	d4102	Kneeling	125	d7601	Child-parent relationships
56	d4103	Sitting	126	d7602	Sibling relationships
57	d4104	Standing	127	d8151	Maintaining preschool educational program
58	d4105	Bending	128	d820	School education
59	d4106	Shifting the body’s center of gravity	129	d8201	Maintaining educational program
60	d4107	Rolling over	130	d835	School life and related activities
61	d4108	Changing basic body position, other specified—from sitting to 4 points/from lying to 4 points/half kn./Turning	131	d880	Engagement in play
62	d415	Maintaining a body position	132	d8800	Solitary play
63	d4152	Maintaining a kneeling position	133	d910	Community life
64	d4153	Maintaining a sitting position	134	d9103	Informal community life
65	d4154	Maintaining a standing position	135	d920	Recreation and leisure
66	d4155	Maintaining head position	136	d9200	Play
67	d4158	Maintaining a body position, other specified—balance in the water/4 points/3 points/half kn./one leg	137	d9201	Sports
68	d420	Transferring oneself	138	d9205	Socializing
69	d4200	Transferring oneself while sitting			
70	d429	Changing and maintaining body position, other specified and unspecified			

ICF-CY—The International Classification of Functioning, Disability and Health: Children and Youth Version.

**Table A5:** Environment Factors (22 categories).

**N.**	**ICF-CY Code**	**ICF-CY Category**
1	e115	Products and technology for personal use in daily living
2	e130	Products and technology for education
3	e150	Design, construction and building products, and technology of buildings for public use
4	e225	Climate
5	e240	Light
6	e250	Sound
7	e260	Air quality
8	e310	Immediate family
9	e315	Extended family
10	e325	Acquaintances, peers, colleagues, neighbors and community members
11	e330	People in positions of authority
12	e355	Health professionals
13	e410	Individual attitudes of immediate family members
14	e420	Individual attitudes of friends
15	e445	Individual attitudes of strangers
16	e450	Individual attitudes of health professionals
17	e455	Individual attitudes of health-related professionals
18	e460	Societal attitudes
19	e5301	Utilities systems
20	e580	Health services, systems and policies
21	e5802	Health policies
22	e585	Education and training services, systems and policies

ICF-CY—The International Classification of Functioning, Disability and Health: Children and Youth Version.

**Table A6:** Body Structures (12 categories).

**N.**	**ICF-CY Code**	**ICF-CY Category**
1	s240	Structure of external ear
2	s250	Structure of middle ear
3	s310	Structure of nose
4	s430	Structure of respiratory system
5	s710	Structure of head and neck region
6	s720	Structure of shoulder region
7	s730	Structure of upper extremity
8	s740	Structure of pelvic region
9	s750	Structure of lower extremity
10	s760	Structure of trunk
11	s770	Additional musculoskeletal structures related to movement
12	s810	Structure of areas of skin
1	s240	Structure of external ear
2	s250	Structure of middle ear
3	s310	Structure of nose
4	s430	Structure of respiratory system
5	s710	Structure of head and neck region
6	s720	Structure of shoulder region
7	s730	Structure of upper extremity
8	s740	Structure of pelvic region
9	s750	Structure of lower extremity
10	s760	Structure of trunk

ICF-CY—The International Classification of Functioning, Disability and Health: Children and Youth Version.
